# MAGE genes encoding for embryonic development in cattle is mainly regulated by zinc finger transcription factor family and slightly by CpG Islands

**DOI:** 10.1186/s12863-022-01034-0

**Published:** 2022-03-18

**Authors:** Bosenu Abera, Hunduma Dinka

**Affiliations:** 1 Department of Applied Biology, School of Applied Natural Sciences, Adama Science and Technology University, P.O. Box 1888, Adama, Ethiopia; 2 Department of Animal Science, College of Agriculture and Natural Resources, Salale University, P.O. Box 245, Salale, Ethiopia

**Keywords:** CpG islands, Embryonic development, MAGE genes, Promoter region, Transcription factor

## Abstract

**Background:**

Melanoma Antigen Genes (MAGEs) are a family of genes that have piqued the interest of scientists for their unique expression pattern. The MAGE genes can be classified into type I MAGEs that expressed in testis and other reproductive tissues while type II MAGEs that have broad expression in many tissues. Several MAGE gene families are expressed in embryonic tissues in almost all eukaryotes, which is essential for embryo development mainly during germ cell differentiation. The aim of this study was to analyze the promoter regions and regulatory elements (transcription factors and CpG islands) of MAGE genes encoding for embryonic development in cattle.

**Results:**

The in silico analysis revealed the highest promoter prediction scores (1.0) for TSS were obtained for two gene sequences (MAGE B4-like and MAGE-L2) while the lowest promoter prediction scores (0.8) was obtained for MAGE B17-like. It also revealed that the best common motif, motif IV, bear a resemblance with three TF families including Zinc-finger family, SMAD family and E2A related factors. From thirteen identified TFs candidates, majority of them (11/13) were clustered to Zinc-finger family serving as transcriptionally activator role whereas three (SP1, SP3 and Znf423) of them as activator or repressor in response to physiological and pathological stimuli. On the other hand we revealed slightly rich CpG islands in the gene body and promoter regions of MAGE genes encoding for embryonic development in cattle.

**Conclusion:**

This in silico analysis of gene promoter regions and regulatory elements in MAGE genes could be useful for understanding regulatory networks and gene expression patterns during embryo development in bovine.

## Background

Reproduction is a complex process that initiated with the production of gametes and leading to formation of the zygote [1]. It involves physiological events that are specific to either the sperm or the oocyte. The regulations of these events are complex processes as they regulated by different genes that are expressed at specific times and locations [2]. These complex processes are mainly driven by large transcriptional changes.

The bovine genome consists of 3 Gb (3 billion base pairs). It contains approximately 22,000 genes of which 14,000 are common to all mammalian species [3]. Promoters are key elements that belong to non-coding regions [4] located adjacently upstream of transcription start sites and control the activation or repression of the genes [5]. Won et al. [6] reported the importance of predicting the promoter region or the transcription start site in investigating the functional roles of gene.

CpG islands are known to regulate gene expression through transcriptional silencing of the corresponding gene. DNA methylation at CpG islands is crucial for gene expression and tissue-specific processes [7]. About half of all CGIs self-evidently contain TSSs, as they coincide with promoters of annotated genes [8]. According to Deaton and Bird [9], most CGIs are sites of transcription initiation including distantly located from annotated promoters.

The melanoma associated antigen (MAGE) genes are conserved in all eukaryotes and lower eukaryotes to 40 genes in humans and mice [10]. They share common MAGE homology domain with high sequence similarity [11]. Some of MAGE genes are ubiquitously expressed in tissues; others are expressed in only germ cells [11]. Flork et al. [10] and Tacer et al. [12] reported that MAGE proteins regulate diverse cellular and developmental pathways and protect the germ-line from environmental stress.

Majority of the MAGE genes are located on the X chromosome and expressed in early spermatogenesis [13]. The MAGE gene can be classified into type I and type II based on their tissue expression pattern [11]. The type I MAGEs have expression restricted to testis and other reproductive tissues [12]. On the other hand, type II MAGEs that have broad expression in many tissues [11, 13]. Several studies reported that MAGE genes play important roles during embryogenesis and germ cell genesis [11–14]. Although studies are conducted on the evolution and biological functions of MAGE genes, there is a limited data on the regulatory mechanisms of this gene during embryo formation in large mammals. Therefore, the aim of this study was to predict promoter and regulatory elements of MAGE genes encoding for embryonic development in cattle *(*Angus*Brahman F1*)* thereby provide basic information for improving reproductive efficiency and fertility in cattle.

## Results

### Identification of TSS and promoter regions of MAGE genes

Promoter region analysis of MAGE genes encoding for embryonic development showed a small variation in the number of TSS where we revealed that 68.42% of the sequences had single TSS (Table 1). The current study also revealed that eight (42.1%) TSSs are located at a distance below -500 bp when checked from the start codon even though TSSs of MAGE genes encoding for embryonic development were mostly located in the upstream region of − 137 to − 1782 bp.

**Table 1:** TSS number and predictive score value for MAGE genes encoding for embryonic development in cattle

Gene Name/ ID	Corresponding promoter region name	No. of TSSs identified	Predictive score value	Distance of best TSSs from ATG
LOC113887351	Pro-MAGEH1	3	0.90,0.97,0.97	-462
LOC113891273	Pro-MAGEF1	2	0.81, 0.98	-335
LOC113887359	Pro-MAGEE2	1	0.90	-910
LOC113879707	Pro-MAGEL2	2	0.84, 1.00	-495
LOC113879741	Pro-NDN	1	0.84	-137
LOC113888173	Pro-MAGE A10-like	1	0.83	-260
LOC113888161	Pro-MAGE A1-like	1	0.96	-850
LOC113888158	Pro-MAGE A9-like	1	0.97	-380
LOC113887980	Pro-MAGE B17-like	1	0.80	-737
LOC113887988	Pro-MAGE B10-like	1	0.98	-986
LOC113888015	Pro-MAGE B16-like	1	0.99	-265
LOC113887630	Pro-MAGE B1-like	1	0.87	-865
LOC113887648	Pro-MAGE B2-like	6	0.83,0.87,0.90,0.91,0.95,0.97	-1782
LOC113887982	Pro-MAGE B5-like	1	0.96	-1626
LOC113887965	Pro-MAGE B4-like	1	1.00	-997
LOC113887799	Pro-MAGE B18-like	2	0.86, 0.94	-851
LOC113887694	Pro-MAGE B3-like	1	0.84	-387
LOC113887472	Pro-MAGE D2-like	2	0.87, 0.90	-1545
LOC113886694	Pro-MAGE A8-like	1	0.99	-907

### Common candidate motifs and associated transcription factors in the promoter regions of MAGE genes

The present analysis discovered five binding motifs from which three motifs (I, III and V) were equally shared (50%) by all MAGE genes encoding for embryonic development in cattle (Table 2). The candidate motif IV was revealed as the best common promoter motif for 66.67% of cattle MAGE genes encoding for embryonic development that serves as binding sites for TFs involved in the expression regulation of these genes.

**Table 2:** Identified common candidate motifs in promoter regions of MAGE genes encoding embryonic development in cattle

Discovered candidate motif	Number (%) of promoters containing each one of the motifs	E-value	Motif width
I	5(27.78)	8.7e-024	46
II	9(50.0)	4.5e-023	49
III	9(50.0)	3.3e-020	41
IV	12(66.67)	6.3e-015	40
V	9(50.0)	8.4e-015	40

**Fig. 1:**
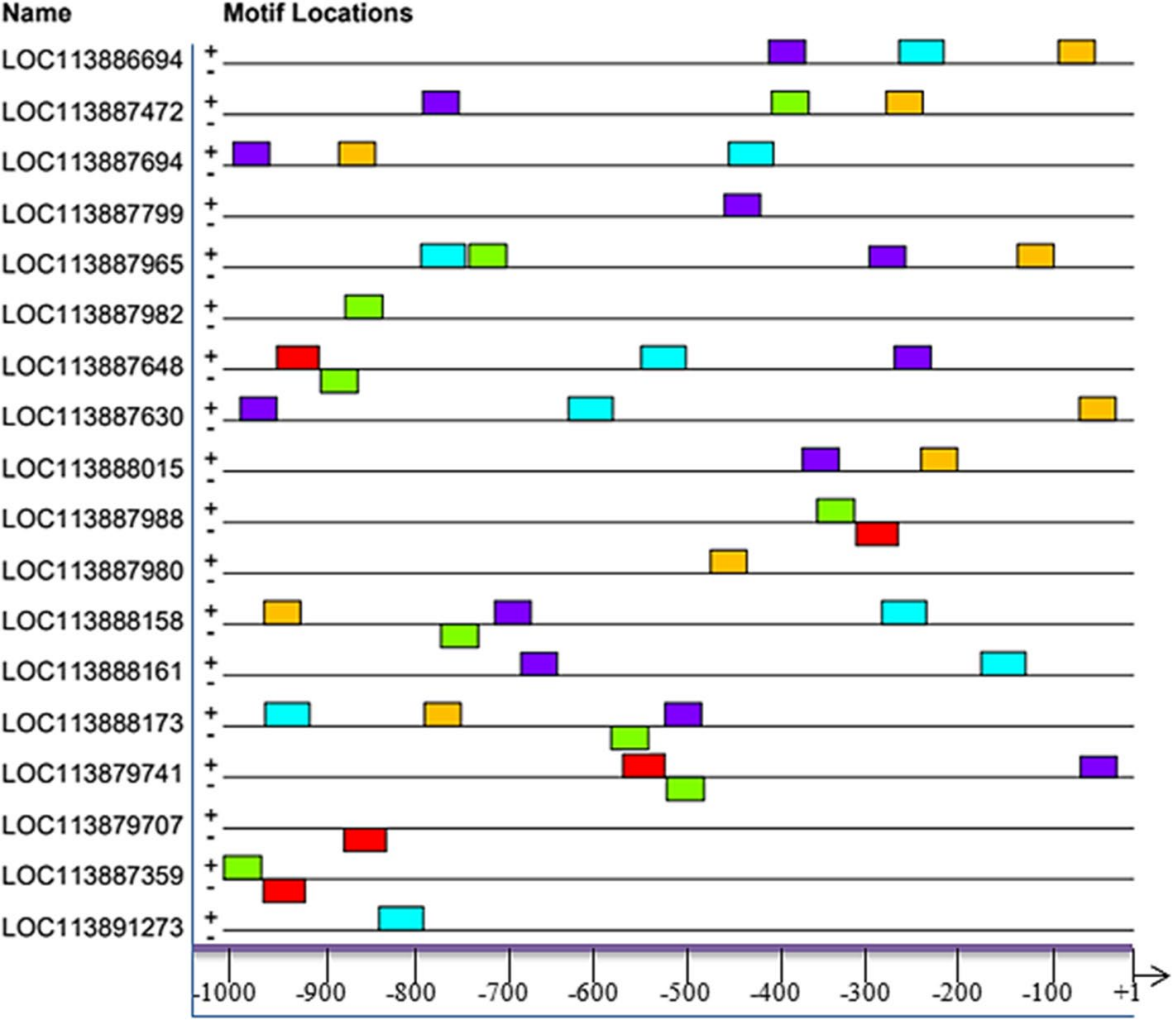
Block diagrams showing the relative positions of candidate motifs in promoter region relative to TSSs. The nucleotide positions are indicated at the bottom of the graph from + 1 (beginning of TSSs) to the upstream 1000 bp in the promoter region for MAGE genes encoding for embryonic development in cattle

The present analysis revealed that majority (61.36%) of the candidate motifs were located and distributed between –700 bp to –200 bp with the reference to the transcription start site region (Fig. 1). The higher distributions of motifs were found in positive than in negative strands.

**Fig. 2:**
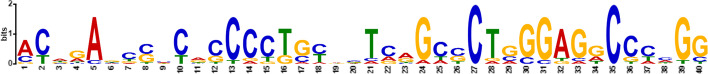
Sequence logos for motif IV, for promoter regions of MAGE genes encoding embryonic development in cattle

To address the information content, MEME created sequence logo for the best common motif, motif IV, which resulted in different characters of motif alignment columns, where the height of the letter represents how frequently that nucleotide is expected to be observed in that particular position (Fig. 2). Motif IV motif was compared with other registered motifs in publically available databases motif in order to explore matched motifs using TOMTOM web application. As a result, motif IV matched with thirteen (13) known motifs found in databases (Table 3).

**Table 3:** The list of TF candidates which could bind to motif IV

TF family	Candidate transcription factors	Regulatory mode	Tissue expression
Zinc finger factors	SP1*(Homo sapiens)*	Dual	Testis and ovary
	EGR1*(Mus musculus)*	Activation	Testis and ovary
	KLF16*(Homo sapiens)*	Repression	Female gonad and testis
	Bcl6b *(Mus musculus)*	Repression	Female gonad and testis
	EGR3*(Homo sapiens)*	Activation	Ovary and testis
	KLF1*(Mus musculus)*	Activation	Bone marrow and spleen
	SP3*(Homo sapiens)*	Dual	Ovary and testis
	KLF5*(Homo sapiens)*	Activation	Testis and placenta
	SP2*(Homo sapiens)*	Activation	Testis and ovary
	Znf423(*Rattus norvegicus)*	Dual	Brain, eye, spleen and heart
	ESR2*(Homo sapiens)*	Activation	Testis and ovary
E2A-related factors	TCF4*(Homo sapiens)*	Activation	Testis, ovary and embryonic tissues expression mostly occurs in the brain
SMAD DNA binding factors	Smad3(Mus musculus)	Activation	brain and ovary

*SP1* Specificity protein 1, *SP2* Specificity protein 2, *SP3* Specificity protein 3, *EGR1* Early growth response 1, *EGR3* Early growth response 3, *KLF16* Kruppel like factor 16, *KLF1* Kruppel like factor 1, *KLF5* Kruppel like factor 5, *ESR2* Estrogen receptor beta, *TCF4* Transcription factor 4, *Znf423* Zinc finger protein 423, Smad3- fusion of Caenorhabditis elegans Sma genes and the Drosophila Mad, Mothers against decapentaplegic homolog 3, *BCL6B* B-cell lymphoma 6, member B *Statistical significance for the binding of given transcription factors to motif IV

The present analysis revealed that the best common motif, motif IV, bear resemblance with three transcription factor families: Zinc-finger family, SMAD family and E2A related factors; where majority (84.6%, 11/13) of them belong to Zinc-finger transcription family. The current study revealed SP1 and SP3 transcription factors activate or repress transcription and have major role in embryonic eye, placenta and skeletal system development as we revealed from Uniprot database.

The findings from UniProt database also revealed that KLF1, KLF5, TCF4 and EGR3 transcription factors were transcriptionally activator and has role in utero embryonic development, intestinal epithelial cell development and nervous system development, muscle spindle development, respectively. Likewise, the transcription factor candidate EGR1 had function in the oocyte maturation.

### Investigation for CpG islands in cattle MAGE genes

To further explore the regulatory elements that are involved in nineteen (19) MAGE genes encoding for embryonic development in cattle, CpG islands were investigated in both promoter and gene body regions using two algorithms. Using Takai and Jones’ algorithm, we found six (6) CpG islands in promoter and five (5) CpG islands in gene body regions (Table 4). In this study, investigation of the CGIs indicated that MAGE genes encoding for embryonic development in cattle have slightly rich CGIs in their promoter and gene body regions.

**Table 4:** CpG islands identified in upstream and gene body regions for 19 MAGE genes in cattle

Gene Name	Promoter region^a^				Gene body region^a^			
	Start site	End site	Length	GC content	Start site	End site	Length	GC content
LOC113879741	503	1047	545	55%	1	953	953	53%
LOC113886694	730	1300	571	63%	197	717	521	59%
LOC113887965	357	1594	1238	59%	-	-	-	-
LOC113887980	656	1251	596	62%	-	-	-	-
LOC113888015	141	702	542	60%	-	-	-	-
LOC113891273	672	1314	643	58%	1	822	822	50%
LOC113889707	-	-	-	-	1	1837	1837	62%
LOC113887351	-	-	-	-	1	536	536	50%

^a^CpG islands are identified by using Takai and Jones’ algorithm searched in 2 kb upstream of ATG and in gene body regions for 19 MAGE genes encoding for embryonic development in cattle

Analysis for CpG islands on both promoter region and gene body region using restriction enzyme *MspI* was also conducted (Table 5). The in silico digestion results revealed more CpG islands in gene body region compared to promoter region; and one gene (LOC113887988) contain two fragment sizes: 113 and 103 bps in gene body region and promoter region, respectively. In the present analysis, about six CGIs and three CGIs were found in gene body region and promoter region, respectively. The results indicated that cattle MAGE genes encoding for embryonic development in cattle are slightly few in CpG islands which is in agreement with the first method, Takai and Jones’ algorithm.

**Table 5:** *MspI* cutting sites and fragment sizes in promoter and gene body regions for 19 MAGE gene sequences encoding for embryonic development in cattle

Sequence name	Gene body region		Promoter region	
	No. & positions of *MspI* cutting sites	Fragment sizes (between 40 and 220 bps)	No. & positions of *MspI* cutting sites	Fragment sizes (between 40 and 220 bps)
LOC113887351	No cut	-	2(1257, 1284)	-
LOC113891273	2(231,727)	-	No cut	-
LOC113887359	1(148)	-	3(171, 1044, 1814)	-
LOC113879707	1(711)	-	1(880)	-
LOC113879741	No cut	-	2(991, 1035)	44
LOC113888173	No cut	-	No cut	-
LOC113888161	No cut	-	No cut	-
LOC113888158	2(627, 678)	51	No cut	-
LOC113887980	2(156, 602)	-	No cut	-
LOC113887988	2(54, 167)	113	3(1332, 1435, 1734)	103
LOC113888015	2(581, 966)	-	No cut	-
LOC113887630	3(127,143,261)	118	No cut	-
LOC113887648	No cut	-	1(229)	-
LOC113887982	No cut	-	1(277)	-
LOC113887965	3(278, 282, 784)	-	No cut	-
LOC113887799	3(124,200,581)	76	3(1229, 1266, 1607)	-
LOC113887694	No cut	-	3(48, 76, 248)	172
LOC113887472	3(184,842,1004)	162	1(1011)	-
LOC113886694	3(437, 615, 666)	51, 178	No cut	-

## Discussion

The retrieved sequence data from NCBI database were used to identify and characterize the promoter regions and regulatory elements of MAGE genes. The findings revealed that promoter region analysis of MAGE genes encoding for embryonic development showed a small variation in the number of TSS. This result is in line Xu et al. [15] who reported that one TSS per gene and that other TSSs arise from errors in transcriptional initiation. However, it is contrary with previous studies on different mammals [16, 17].

The current study also revealed that TSSs of MAGE genes encoding for embryonic development was mostly located in the upstream region of -137 to -1782 bp. This result is in agreement with Mu et al. [18] who reported transcriptional initiation site location of -515 bp for ovine *DKK1* gene and Pokhriyal et al. [19] who reported TSS location at 235 bp, 156 bp and 92 bp for BICP0, BICP4 and BICP22 in bovine genes, respectively.

The current analysis discovered multiple binding motifs for MAGE genes, which is significant to find all possible binding motifs for the same TF and co-factor binding motifs [20]. Likewise, the analysis revealed multiple binding sites in the promoter region of candidate motifs, which could be used to strengthen binding interactions and different regulatory effect [21]. The majority of candidate motifs in the promoter regions of MAGE genes are located and distributed between –700 bp to –200 bp with reference to transcription start site region. This is in agreement with Halees [22] who reported that majority of motifs are located immediately upstream of a TSS. The candidate motifs were highly distributed in the positive strands than negative strands.

The present analysis revealed that the best common motif, motif IV, bear resemblance with three transcription factor families: Zinc-finger family, SMAD family and E2A related factors; where majority (84.6%, 11/13) of them belong to Zinc-finger transcription family. This is in agreement with Samuel and Dinka’s [17] finding who reported zinc finger family transcription factors are the main regulatory element for olfactory receptor in cattle. Adryan and Teichmann [23] showed that zinc finger transcription factors are strongly represented early in embryonic development and they are typically regulate gene expression by binding to specific DNA sequences via their DNA-binding zinc finger domains [24].

The current findings revealed that the observed SP1 and SP3 transcription factors have dual regulatory function and have major role in embryonic eye, placenta and skeletal system development. This is in close agreement with previous studies on the transcription factors Sp1 and Sp3 expression and regulatory functions in mammalian cells [25–27]. Similarly, findings from Uniprot database revealed that transcription factors KLF1, KLF5, TCF4 and EGR3 are transcriptionally activator and have role in different embryonic tissue development. This result is in agreement with Chen et al. [28] and Wang et al. [29] who reported that Krüppel-like factor families are important role in maintaining embryonic stem cells.

It has been reported that CGIs are highly involved in gene regulatory processes [9]. In this study, investigation of the CGIs indicated that MAGE genes encoding for embryonic development in cattle have slightly rich CGIs in their promoter and gene body regions. The in silico digestion results also revealed slightly rich in CpG islands in cattle MAGE genes encoding for embryonic development which is in agreement with the first method, Takai and Jones’ algorithm. Similar findings are reported by Reik and Walter [30]. The author reported that the CpG islands associated with the MAGE genes have a CpG-rich region of 300–650 bp long at their 5’end. CpG islands are often associated with the promoters of most house-keeping genes and many tissue-specific genes, and thus have important regulatory functions and can be used as gene markers [31]. However, Samuel and Dinka [17] reported poor CGIs using *MspI* enzyme digestion for cattle olfactory receptor genes.

The present in silico study analyzed promoter and regulatory elements of MAGE genes in cattle using different algorithms. However, due to various physiological and biological functions as well as broad expression of MAGE genes in tissues, we are not sure to fully recommend the direct role of MAGE genes in embryonic development. Thus further in vitro or in vivo experiment should validate the findings. It is normal that validation is important for in silico study approach or other computational based approach. Thus the limitation of present study is that it is in silico analysis which requires confirmation by experimental validation.

## Conclusions

Identification and characterization of promoter regions of MAGE genes encoding for embryonic development in cattle is essential for understanding the regulatory mechanisms that control its expression. The current finding showed that regulatory elements found in the promoter region of MAGE genes may play direct roles in the gametogenesis process and then in embryo development. The current results would assist animal scientists in boosting cattle reproduction efficiency. However, further experimental studies will be necessary to validate the role of identified transcription factors and their common binding sites in the regulation of MAGE genes encoding for embryonic development in cattle.

## Methods

### Selection/retrieval of MAGE gene from NCBI

Distinct coding sequences belonging to MAGE gene family were retrieved from NCBI database via web-server https://www.ncbi.nlm.nih.gov. The MAGE genes of Angus*Brahman FI hybrid cattle breed were extracted from UOA_Brahman_1 genome assembly and they were further characterized using genomic resources UniProt (https://www.uniprot.org). Duplicate and nonfunctional sequences were discarded from analysis. In this analysis, from a total of twenty one (21), nineteen (19) representative functional protein coding genes, with single exons, that have ORF were considered. Multi-exon genes were excluded from analysis as they have variable promoter region and produce different protein isoforms at different promoters [32, 33] that makes difficult to predict regulatory elements.

### Determination of transcription start sites and promoter regions for MAGE genes

In order to determine TSSs of each gene, minimum of 1 kb upstream of the start codon were excised from each gene [34]. The retrieved segments were fitted to Neural Network Promoter Prediction (NNPP version 2.2) by setting the minimum standard predictive score (between 0 and 1) with a cut off value of 0.8 [35]. This tool helps us to locate the possible TSSs within the sequences upstream of the start codon. For sequences having multiple TSSs, the TSS with the highest prediction value was considered as statistically significant and accurate. The promoter regions were determined 1 kb region upstream of each TSS as previously described by Michaloski et al. [36] for mouse odorant and vomeronasal receptor (V1R) genes.

### Identification of common candidate motifs and transcription factors (TFs)

The predicted promoter sequences of MAGE genes were analyzed using the MEME((Multiple Em for Motif Elicitation) version 5.3.3 searches [37] to discover common candidate motifs that serve for binding sites of transcription factors regulating expression of MAGE genes. The MEME output in HTML format, significant motif, was submitted to TOMTOM [38] for TF prediction. The TOMTOM compared one or more motifs against a database of known motifs and produce an alignment for each significant match and produced LOGOS with *p*-value and q-value [39].

### Search for CpG islands

In order to identify CpG islands in the upstream of MAGE genes, 2 kb sequences upstream of the start codon were used from each gene. The body regions of MAGE genes were also analyzed. The CpG islands were studied using two algorithms. The first algorithm, Takai and Jones algorithm with GC content ≥ 55%, Observed CpG/Expected CpG ratio ≥ 0.65, and length ≥ 500 bp was used [40]. This analysis was done via CpG island searcher program (CpGi130) accessible at web link http://dbcat.cgm.ntu.edu.tw/. Secondly, the offline tool, CLC Genomics Workbench version 5.5.2 (http://clcbio.com, CLC Bio, Aarhus, Denmark) was used for searching the restriction enzyme *MspI* cutting sites (with fragment sizes between 40 and 220 bp parameters). Searching for *MspI* cutting sites is relevant for detection of CGIs and it recognizes CCGG sites [41].

## Data Availability

The datasets used and/or analysed during the current study are available from the corresponding author on reasonable request.

## Abbreviations

TSS: Transcription Start Site
TF: Transcription factors
MAGE: Melanoma associated antigen
NNPP: Neural Network Promoter Prediction
ORF: Open Reading Frame
NCBI: National Center for Biotechnology Institute
